# Registration of noncommercial randomised clinical trials: the feasibility of using trial registries to monitor the number of trials

**DOI:** 10.1186/1745-6215-13-140

**Published:** 2012-08-20

**Authors:** James Raftery, Eleanor Fairbank, Lisa Douet, Louise Dent, Alison Price, Ruairidh Milne, Tom Walley

**Affiliations:** 1 University of Southampton, Southampton, UK; 2 University of Liverpool, Liverpool, UK

**Keywords:** Trial registration, Noncommercial randomised clinical trials

## Abstract

**Background:**

A 2003 survey suggested the number of noncommercial trials in the UK was declining. Formation of the NIHR in 2006 and increased research spending by the Department of Health may have increased the number of noncommercial trials but no data are available.

**Methods:**

Available data on UK noncommercial trials (were obtained from the two relevant registries: ISRCTN register for the UK, and US ClinicalTrials.gov. Data on each trial were sorted by start year, and compared with the: 2003 survey, and UKCRN portfolio database from 2007.

**Results:**

The number of UK noncommercial trials registered rose from 25 in 1990 to 188 in 1999, peaked at 533 in 2003, and fell back to 334 in 2009. Total trials registered was similar to but slightly above those in the 2003 survey up to 1998, then rose sharply to 2002 before falling to 2007. From 2007 to 2009 the number registered to start each year was similar to but slightly above the UKCRN database. Less than 10% of UK noncommercial trials registered with ClinGov for most years before 2005, but this rose to 35% by 2009.

**Conclusions:**

For the periods of overlap, trial registration data provide fairly similar totals to other sources on the number of noncommercial trials starting each year. The rise and fall in the number of trials registered between 1999 and 2007 was due to those registered in the ISRCTN database as funded by NHS Trusts. After 2007, the number of trials registered as funded by NHS Trusts has fallen in the ISRCTN register but these trials may have migrated to the US ClinGov register. The total number of noncommercial trial starts, excluding those funded by NHS Trusts, has been upward since around 2002. By 2009 the two main funders were NIHR and charities. Feasibility of using registration data to monitor the number of noncommercial trials has been demonstrated but is complicated by the use of two registers and difficulties in accessing the data. We recommend an annual report on the number of noncommercial trials registering each year.

## Background

Concerns have been raised about a reduced number of clinical trials conducted in the UK, both commercial and noncommercial [1]. However, the Medicines and Healthcare Products Regulatory Agency – which authorises trials of medicines – has put the number of trials at a steady 1,200 per year from 2002 to 2009, split roughly into 75% commercial and 25% noncommercial [2,3].

Chalmers and colleagues sounded an alarm in 2003 that the number of noncommercial trials in the UK was declining, based on a survey of the main funders [4]. This survey showed around 150 trials starting in 1997, falling to 40 in 2002. From 2007 the UK Clinical Research Network (UKCRN) portfolio database on trials being carried out in the National Health Service (NHS) put the number of noncommercial trials starting each year in the UK at around 300 [5].

Registration of clinical trials has become increasingly common due to statutory requirements in the USA [6] and for publication in medical journals [7]. The revised 2008 Declaration of Helsinki states that ‘Every clinical trial must be registered in a publicly accessible database before recruitment of the first subject’ [6].

Although registration is not compulsory in the UK, clinical governance arrangements from 2004 have required registration [8].

UK triallists have registered with either the US Clinical Trials Government Register (ClinGov) or the International Standard Randomised Controlled Trials Number register (ISRCTN). ClinGov, run by the US National Library of Medicine, was the first online registry for clinical trials and remains widely used, not only for US-based trials. Its origins lie with the Health Omnibus Programs Extension Act of 1988 (Public Law 100–607), which mandated the development of a database of clinical trials of treatments for AIDS. The registry was expanded under the Food and Drug Modernization Act of 1997 (Public Act 105–115) [9].

The alternative to the US registry is the International Standard Randomised Controlled Trial Number registry, established in 1998. The UK register ISRCTN.org is a primary partner in the World Health Organisation platform, run as Current Controlled Trials (CCT), part of Springer Science + Business Media [9]. CCT provides free and open access to information about registered randomised clinical trials. From 2004 the UK Department of Health, under its research governance framework, contracted with CCT to register trials funded by the HTA programme and ‘those funded at trust level by the NHS R&D Support Funding stream’ [10]. ISRCTN numbers were allocated to all trials funded by previous NHS R&D programmes, and to ‘own account’ trials funded by NHS Trusts. CCT evidence to the House of Commons noted that less than 5% of the trials registered on the ISRCTN registry were funded by industry [10]. Industry-funded drug trials register elsewhere. From 2004 all new and ongoing trials involving a Clinical Trial Authorisation from the Medicines and Healthcare Products Regulatory Agency had to register with the European database, EudraCT, which is confined to drugs and is confidential.

Owing to it being the first international register, some UK trials registered with ClinGov before the ISRCTN register was established in 1998. The US register allocates unique eight-digit numbers with the prefix NCT, while the ISRCTN registers allocate eight-digit identifiers with the prefix ISRCTN. Both the US and UK registers require largely the same data but with two key differences: source of funding is a separate obligatory field in ISRCTN but not in ClinGov. The other difference is that ClinGov provides free registration while ISRCTN requires a registration fee (£200 in 2012). This fee is met by the UK Department of Health for trials funded by it or partner agencies but not for other trials. The CCT website provides online access to both the ClinGov and ISRCTN registers via a meta-register. ClinGov had 62,734 trials registered in November 2011 and ISRCTN had 10,153. We used the meta-register to identify UK trials (defined to include all trials with at least one recruitment centre in the UK) by register, to estimate the total number of noncommercial trials over time, and as far as possible to subdivide these trials by funder type.

Our aim was to establish the number of UK-based noncommercial randomised clinical trials registered each year, with cross-checks against other sources, specifically Chalmers data for 1990 to 2002 [4] and the UKCRN portfolio database for 2007 to 2009 [5].

## Methods

We compiled a dataset of noncommercial randomised clinical trials due to start in the UK in any year between 1990 and 2009 from two trial registration databases, the ISRCTN register [11] and the US register ClinicalTrialsGov [9]. Data were purchased from CCT on the trials in the ISRCTN database, via a Wessex Institute small research grant. Data on randomised clinical trials with at least one UK centre registered with ClinGov were downloaded from the website with noncommercial trials identified using sponsors. Data were available from 1990 in both databases due to the forerunner of ClinGov having been established in 1988 and due to data on clinical trials from the National Research Register [12], a prior database of all Department of Health-funded research projects, having been backloaded onto ISRCTN. This enables comparison with Chalmers and colleagues’ results, whose survey covered trials commencing each year from 1990 to 2003 [4].

Criteria for eligibility were description as a randomised trial, a start date, a sponsor and, if available, a funder. Duplicates were excluded and the records sorted by year of proposed start of recruitment. Data extraction was carried out by EF, LDo, AP and LDe. Classification of funders was carried out independently by this group and by JR, and differences were reconciled through further web searches and discussion. The number of trials identified was compared with Chalmers and colleagues’ 2003 survey [4] and with the UKCRN portfolio database for 2007 to 2009 [5].

## Results and discussion

A total of 4,569 eligible records were identified as eligible after exclusion of duplicates.

**Figure 1:**
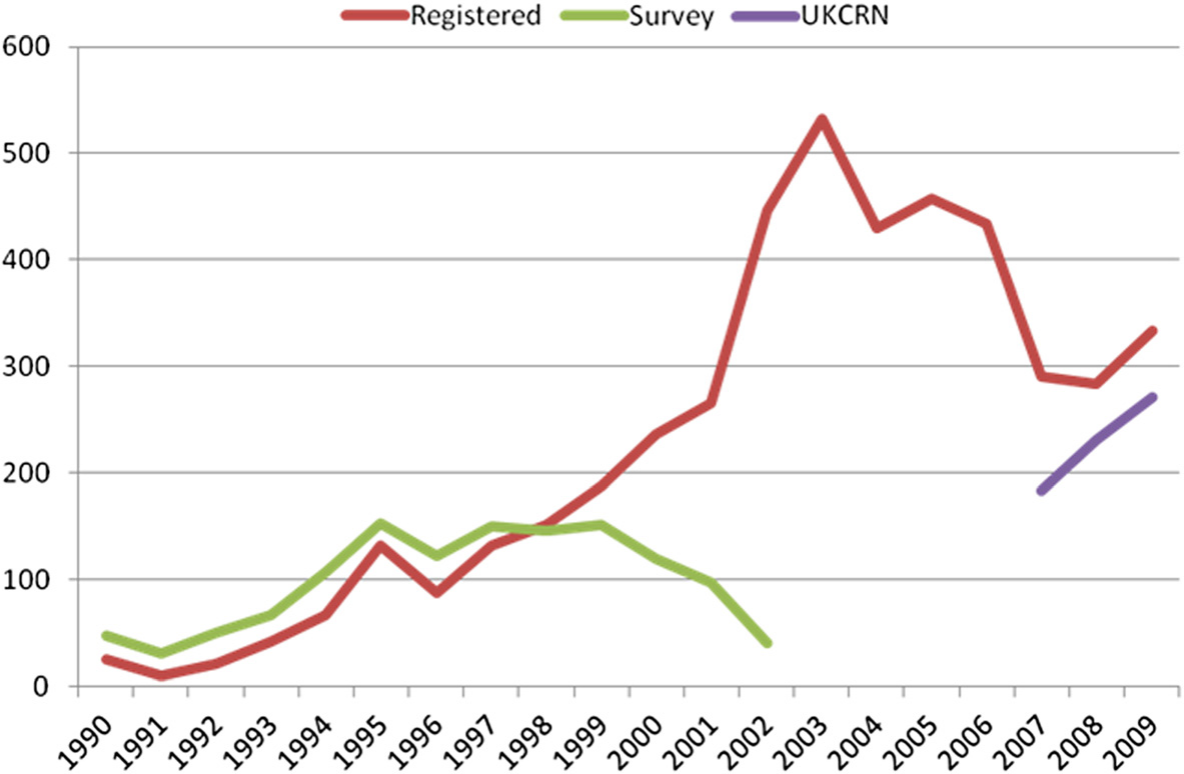
Noncommercial trials registered, by year, with numbers in 2003 survery and UK CRN portfolio.

The number of noncommercial trials registered rose from 25 in 1990 to 188 in 1999, and to a peak of 533 in 2002, before falling back to 334 in 2009 (Table 1). Figure 1 shows that the total number of trials registered was similar to but slightly above those in Chalmers and colleagues’ survey of funders for first part of the period (up to 1998), but above Chalmers and colleagues’ results for 1999 to 2003. For 2007 to 2009, the number registered to start each year was similar to but slightly above the UKCRN database.

**Table 1:** Number of UK noncommercial trials registered and distribution by ClinGov and ISRCTN, 1990 to 2009

**Year**	**Number of UK trials registered**	**% registered with ClinGov**	**% registered with ISRCTN**
1990	25	4	96
1991	10	0	100
1992	21	25	75
1993	42	16.7	83.2
1994	57	7.6	92.4
1995	132	7.6	92.4
1996	88	6.9	93.1
1997	132	8.3	91.7
1998	151	15.1	84.9
1999	188	8.5	91.5
2000	236	5.5	94.5
2001	265	13.6	86.4
2002	447	3.6	96.4
2003	533	8.3	91.7
2004	430	9.3	90.7
2005	458	13.5	86.5
2006	434	15.7	84.3
2007	291	31.3	68.7
2008	284	31.7	68.3
2009	334	35	65
Total	4,568		

ClinGov, US Clinical Trials Government Register; ISRCTN, International Standard Randomised Controlled Trial Number.

Table 1 shows where these trials registered. Less than 10% of UK trials registered with ClinGov for most years before 2005 but the proportion rose thereafter to reach 35% by 2009.

The source of funding is shown in Table 2. Since ClinGov does not include a field for sources of funding, the funding for trials so registered was classed as unknown. Owing to the increase in the proportion of UK trials registering with ClinGov, unknown funding accounted for the largest share – 35% in 2009.

**Table 2:** Noncommercial randomised UK clinical trials, number registered each year by funder, 1990 to 2009

**Funder**	**Charity**	**MRC**	**NHS R&D**	**NHS Trusts**	**Other**	**Unknown**	**Total**
1990	14	7		3		1	25
1991	3	6		1			10
1992	3	5	8			5	21
1993	7	10	15	3		7	42
1994	7	16	36	3		5	67
1995	22	13	76	10	1	10	132
1996	8	17	57			6	88
1997	16	18	78	8	1	11	132
1998	23	19	70	16	1	22	151
1999	15	38	85	33	1	16	188
2000	41	24	73	84	2	13	237
2001	41	12	39	134	2	37	265
2002	56	13	27	329	6	16	447
2003	54	10	32	388	5	44	533
2004	63	11	38	269	8	41	430
2005	77	15	40	252	11	63	458
2006	85	15	30	222	11	71	434
2007	75	16	47	55	7	91	291
2008	69	12	64	36	12	91	284
2009	59	15	89	45	8	118	334
Grand total	738	292	904	1,891	76	668	4,569

Charity includes Cancer Research UK and a small number of educational grants from pharmaceutical companies. Other includes a small number of investigator-funded projects and PhD studies. National Health Service (NHS) R&D includes all NHS-funded projects with the exception of those randomised controlled trials with funder indicated as an NHS Trust. Unknown indicates that either the funding field was missing (almost all instances) or had not been completed (rare). MRC, Medical Research Council.

**Figure 2:**
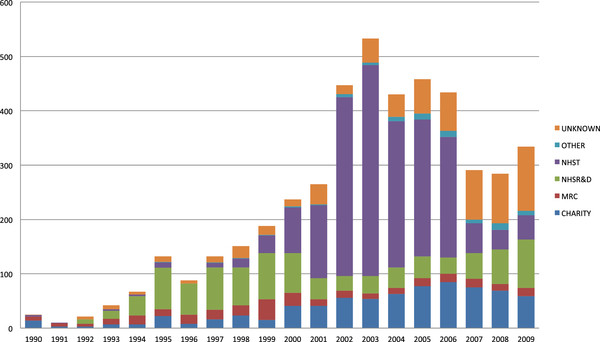
Number of UK noncommercial randomised trials registered by funding source, 1990 to 2009.

The sharp rise in the number of registered trials from 1999 to 2003 was due almost entirely to those registered as funded by NHS Trusts (Figure 2). The proportion so registered jumped from 17% in 1999 to 72% in 2003 before falling back to 18% in 2007. By 2009 the National Institute for Health Research (NIHR), formerly NHS R&D, was the largest funder (25%), followed by the charities (17%), of which Cancer Research UK was the largest followed by the Arthritis Research Council, the British Heart Foundation and the Wellcome Trust.

The total number of UK noncommercial trials registered was similar to those of Chalmers and colleagues for 1993 to 1998 [4] and to the UKCRN portfolio database for 2007 to 2009 [5]. Between 1998 and 2002, the second period of overlap with Chalmers and colleagues, the total registered rose while the total in Chalmers and colleagues’ survey fell.

The number of noncommercial trials registered over the past 20 years has increased from under 200 before 2000 to around 300 per annum from 2007 to 2009. A rapid rise in the total from 1996 to a peak in 2003 of over 500 and the subsequent fall to 2007 was evident. This was due mainly to trials registered as funded in ISRCTN by NHS Trusts, which have not previously been identified as major funders. No new funding became available to NHS Trusts around this time. Governance of trials increased after the EU Directive of 1999, which became UK law in 2004 with the Medicines for Human Use (Clinical Trials) Regulations [11]. The strong encouragement of trial registration around that time in the UK may explain the rise in the number of trials registered.

The decline in the number of trials registered as funded by NHS Trusts after 2003 might be linked to changes in funding. Each hospital had a largely notional self-declared research fund that had historically covered a range of activities, including the Service Increment for Teaching and Research, Special Health Authorities, ‘tasked’ monies for general practice research and ‘own account’ research [13-15]. In 2006/07 some £500 million or some 80% of the NIHR (NHS R&D up to 2006) budget was hospital based [16]. These funds were successively known as Culyer Budget 1, infrastructure support, Priorities and Needs Funding, and Support for Science [17]. Some of what was termed ‘own account’ research was funded by these funds. These considerable funds were withdrawn over three transition years from 2006/07, reducing the scope for NHS Trusts to fund research [18]. The decline in the number of trials registered as funded by NHS Trusts from 2007 is plausibly linked to this change in funding.

The rise in the proportion of UK trials registering with ClinGov rather than ISRCTN is notable. This rose from 10 to 15% before 2007 to over 30% from 2007 to 2009. Although the ISRCTN charges for registration, these charges are met by the Department for Health for trials funded by the main funders, specifically the NIHR, Medical Research Council and the charities. It seems likely that those trials registering with ClinGov were from other funders, such as ‘own account’ trials in trusts. Whatever the reason, the rise in the use of ClinGov was unfortunate because of its lack of a field on the funding of the trial. This means that in 2009 the largest group of trials were those with unknown funding. If registration data are to be of use in monitoring trends in noncommercial trials the source of funding must be known.

## Conclusions

Overall, for the periods of overlap with other sources, trial registration data provide fairly similar results, except after 1998 when a rapid rise and fall in the number of trials registered as funded by NHS Trusts occurred. We have suggested that these changes may have been due to ‘own account’ trials, with changes in governance leading to a rise in registration and changes in funding accounting for the fall. How many of the trials registered as funded by NHS Trusts report their results remains to be seen.

As the number of trials registered as funded by NHS Trusts has fallen, the number of UK randomised trials registering with ClinGov has increased sharply, so that 35% of UK trials registering in 2009 were with ClinGov. These were probably largely those trials previously registered as funded by NHS Trusts, but this cannot be established without more detailed investigation.

Leaving aside trials registered as funded by NHS Trusts, the overall trend has been upward since around 2002. By 2009 the two main funders were the NIHR and charities. The role of the Medical Research Council, already reduced, seems likely to shrink as NHS trials become the responsibility of the NIHR. Owing to the increased use of ClinGov by 2009, however, the largest group of trials registered in that year had no data on the funder. Further work to understand how these trials are being funded and registered with ClinGov seems an obvious priority.

The feasibility of using registration data to monitor the number of noncommercial trials is complicated by the use of two registers, and difficulties in accessing the data. However, it is feasible to compile these data. We recommend annual reporting on the total number of noncommercial trials registering each year.

Finally, we acknowledge that the number of randomised trials says nothing about the quality or size of such trials. One good or large trial may be more valuable than several poor or much smaller trials. Assessing the quality and size of trials, however, are topics for further study.

## Abbreviations

CCT: Current Controlled Trials; ClinGov: US Clinical Trials Government Register; ClinicalTrialsGov: service provided by US National Institutes of Health; ISRCTN: International Standard Randomised Controlled Trial Number; NHS: National Health Service; NIHR: National Institute for Health Research; UKCRN: UK Clinical Research Network.

## Competing interests

The authors declare that they have no competing interests.

## Authors' contributions

The original idea originated from JR, RM and TW and was refined in discussion by all of the authors. The data were compiled by EF, LDo, LDe and AP. The paper was drafted by JR and EF with contributions from all the authors. All authors gave final approval to the article.

## References

[B1] Gallan A Number of clinical trials done in UK fall by two thirds after the EU directive BMJ 2009 33 1052 10.1136/bmj.b1052 19286745

[B2] Press Release: New Report Examines Obstacles to Non-commercial Clinical Trials [ http://www.mhra.gov.uk/PrintPreview/PressReleaseSP/CON041316].

[B3] MHRA Freedom of Information Request [ www.mhra.gov.uk/home/groups/es-foi/…/foidisclosure/con2031623.pdf].

[B4] Chalmers I Rounding C Lock K Descriptive survey of non-commercial randomised controlled trials in the United Kingdom, 1980–2002 Br Med J 2003 327 1017 1019 10.1136/bmj.327.7422.1017 14593034 PMC261654

[B5] UKCRN Portfolio Database [ http://public.ukcrn.org.uk/search/]

[B6] Bian ZX Wu TX Legislation for trial registration and data transparency Trials 2010 11 64 10.1186/1745-6215-11-64 20504337 PMC2882906

[B7] De Angelis CD Drazen JM Frizelle FA Haug C Hoey J Horton R Kotzin S Laine C Marusic A Overbeke AJ Schroeder TV Sox HC Van Der Weyden MB Is this clinical trial fully registered? A statement from the International Committee of Medical Journal Editors Ann Intern Med 2005 143 146 148 16027458 10.7326/0003-4819-143-2-200507190-00016

[B8] NHS Research Governance for Health and Social Care: Second Edition [ http://www.dh.gov.uk/en/Publicationsandstatistics/Publications/PublicationsPolicyAndGuidance/DH_4108962]

[B9] ClinGov [ http://clinicaltrials.gov/ct2/]

[B10] Registration of Randomised Clinical Trials and Public Access to Findings. Further Information 2009 London: Department of Health [ http://www.controlled-trials.com/]

[B11] ISRCTN MetaRegister of Current Controlled Trials [ www.controlled-trials.com]

[B12] National Research Register [ http://www.nihr.ac.uk/Pages/NRRArchive.aspx]

[B13] House of Commons Health Committee 2004. Evidence by CCT [ http://www.google.co.uk/#hl=en&cp=36&gs_id=3s&xhr=t&q=CCT+evidence+house+commons+health+committee&pf = p&sclient = psyb&source = hp&pbx = 1&oq = CCT + evidence + house + commons + health + co&aq = 0w&aqi = qw1&aql = &gs_sm = &gs_upl = &bav = on.2,or.r_gc.r_pw.,cf.osb&fp = 15bc013397bd8a17&biw = 1008&bih = 841]

[B14] Stephen H Shyama K Bryony S Nicholas M Who Needs What from a National Health Research System: Lessons from Reforms to the English Department of Health’s R&D System Health Research Policy and Systems 2010 8 11 10.1186/1478-4505-8-11 20465789 PMC2881918

[B15] Shergold M Grant J Freedom and need: the evolution of public strategy for biomedical and health research in England Health Res Policy Systems 2008 6 2 10.1186/1478-4505-6-2 PMC2259354 18230124

[B16] Culyer AJ Funding Research in the NHS Discussion Paper 1994 York: University of York

[B17] NIHR Annual Report 2009/10 Embedding Health Research [ http://www.nihr.ac.uk/files/pdfs/400891_NIHR_AnnualReport2010_acc3.pdf]

[B18] Department of Health. 2008/09 NHS R&D Transitional Funding [ http://www.dh.gov.uk/en/Aboutus/Researchanddevelopment/AtoZ/NationalNHSRDfunding/index.htm]

